# Acute Lymphocytic Myocarditis in a Young Male Post-COVID-19

**DOI:** 10.1155/2023/7646962

**Published:** 2023-06-22

**Authors:** Mintje Bohné, Sebastian Bohnen, Stephan Willems, Karin Klingel, Dietmar Kivelitz, Edda Bahlmann

**Affiliations:** ^1^Department of Cardiology, Asklepios Clinic St. Georg, Hamburg, Germany; ^2^Cardiopathology, Institute for Pathology, University Hospital Tübingen, Tübingen, Germany; ^3^Department of Radiology, Asklepios Clinic St. Georg, Hamburg, Semmelweis University, Budapest, Asklepios CampusHamburg, Germany

## Abstract

**Background:**

Lymphocytic myocarditis is a rare form of myocarditis, associated with a high mortality rate due to a high risk of sudden cardiac death. Lymphocytic myocarditis might present as a relevant extrapulmonary manifestation after coronavirus disease 2019 (COVID-19) infection. *Case presentation*. We report a case of a 26-year-old male with lymphocytic myocarditis, presenting with a 1-month history of increasing fatigue, palpitations, and shortness of breath. Eight weeks before, he was tested positive for SARS-CoV-2. He had received 2-dose schedule of the COVID-19 mRNA vaccine Comirnaty® (BioNTech/Pfizer) 6 months prior to his admission. Diagnostic work-up by echocardiography and cardiac magnetic resonance (CMR) imaging demonstrated a severely reduced left ventricular function and a strong midmyocardial late gadolinium enhancement (LGE). Histology and immunohistology of the endomyocardial biopsies revealed an acute lymphocytic myocarditis. Immunosuppressive therapy with a steroid taper in combination with azathioprine 300 mg/day was initiated. The patient was equipped with a LifeVest®. On day 17, a non-sustained ventricular tachycardia was documented. Follow-up CMR imaging after 3 months showed a slightly improved systolic left ventricular function, and a strong LGE was still detectable.

**Conclusions:**

The case highlights the significance of recognizing lymphocytic myocarditis correlated to COVID-19. It is important to be vigilant also of a later presentation of cardiomyopathy in patients diagnosed with COVID-19 due to high mortality without immediate support.

## 1. Background

Myocarditis is an inflammatory disease of the heart muscle with myocyte degeneration and necrosis of non-ischemic origin, which is diagnosed by established histological [1] and immunohistological criteria [2]. The inflammation of the heart may be caused by different infectious agents, systemic diseases, drugs, and toxins, but viral infections are recognized as the most common [2].

## 2. Case Presentation

### 2.1. Timeline

**Figure 1:**
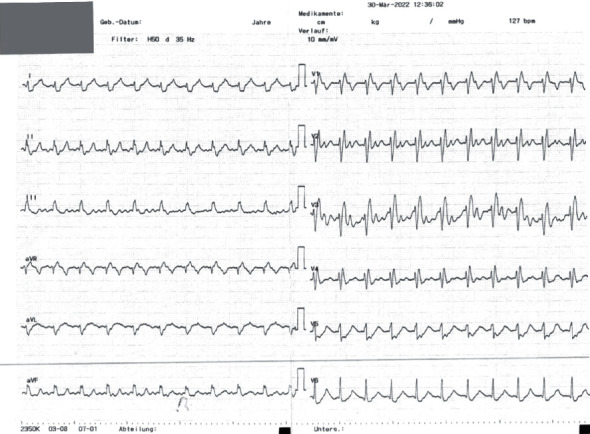
Electrocardiogram demonstrating atrial tachycardia with 127 beats per minute and a right bundle branch block configuration.

**Figure 2:**
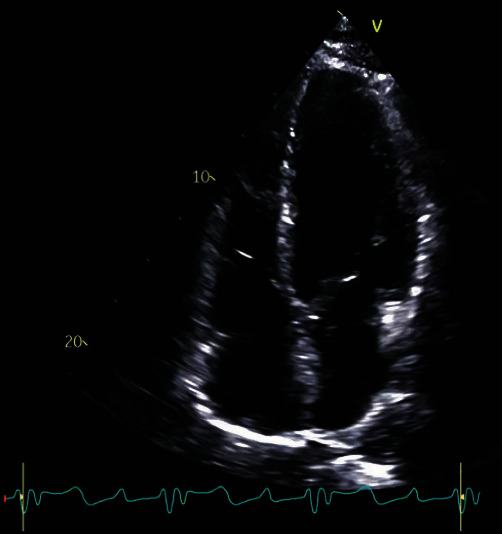
Transthoracic echocardiography showing four chamber cardiac view.

**Figure 3:**
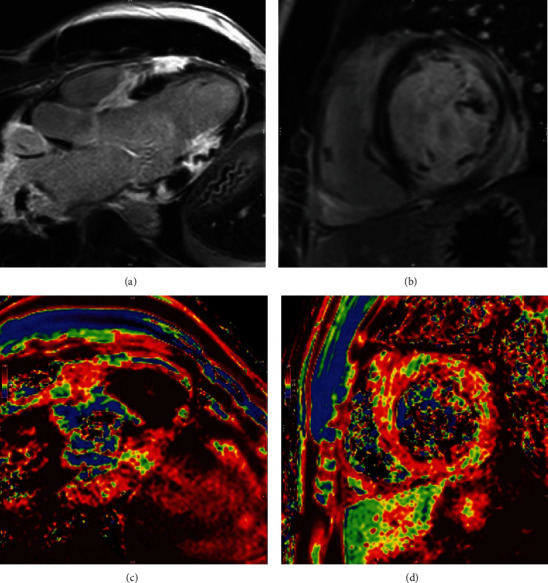
CMR imaging study showing severe LV dysfunction and signs of acute myocarditis. (a and c) Three-chamber views, (b and d) short axis views. (a and b) LGE images with severe subepicardial to transmural LGE inferolateral and anterolateral. (c and d) Native *T*1-maps with focal increased native *T*1 values in LGE positive regions.

**Figure 4:**
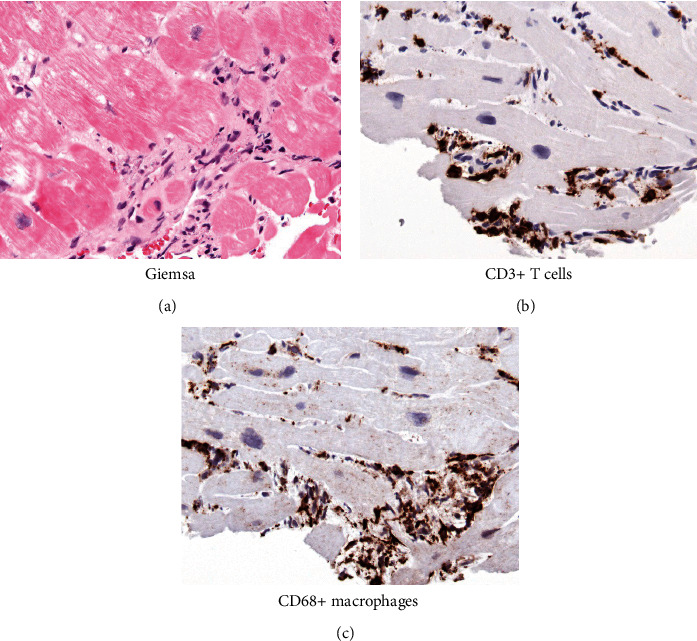
Histopathological images. (a) Histological findings in the endomyocardial biopsy showing active lymphocytic myocarditis with necrosis and areas of organization (Giemsa), (b) CD3 and T-cells, and (c) CD 68 and macrophages.

**Figure 5:**
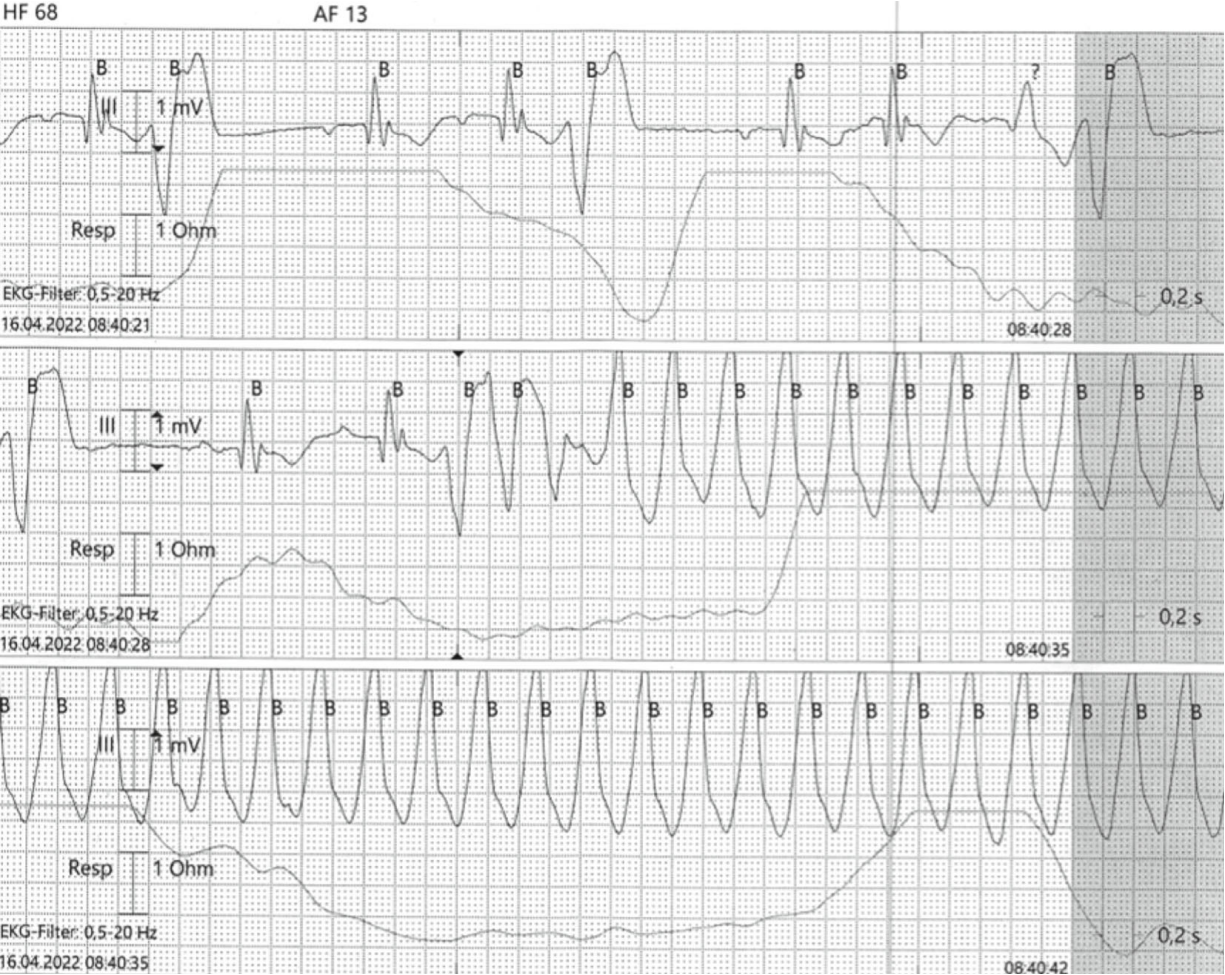
Electrocardiogram demonstrating non-sustained ventricular tachycardia and frequent premature ventricular complexes.

We report a 26-year-old, previously healthy male admitted to our emergency unit by his family doctor with a 1-month history of increasing fatigue, palpitations, and shortness of breath. The patient had prior coronavirus disease 2019 (COVID-19) 2 months before his admission. He had received a 2-dose schedule of the COVID-19 mRNA vaccine Comirnaty® (BioNTech/Pfizer) latest 6 months ago. Two weeks prior to his admission, a pulmonary embolism could be ruled out by a thoracic computed tomography scan but was noticeable for a cardiomegaly, residual pulmonary infiltrates, and increased bihilar lymphatic tissue. Physical examination, besides an increased respiratory rate of 17/minute and a heart rate of 123 bpm, revealed no pathologies, in particular no peripheral leg edema. The arterial blood pressure was 124/84 mmHg. Blood analysis was noticeable for elevated cardiac markers with a Troponin I of 103 ng/l (ref: <34 ng/l), *D*-Dimer of 1.3 mg/l (ref: <0.5 mg/l), and an N-terminal prohormone of brain natriuretic peptide of 2421 ng/l (ref: <125 ng/l). His blood count showed normal hemoglobin concentration, eosinophilic and platelet count, and no leukocytosis. A first nasopharyngeal swab for SARS-CoV-2 RNA detection was negative. Electrocardiogram showed an atrial tachycardia and a right bundle branch block (Figure 1). Transthoracic echocardiogram demonstrated a severely impaired biventricular systolic function. Pericardial effusion could be ruled out as well as a valvular heart disease (Figure 2). After excluding intraventricular thrombus formations, electric cardioversion could restore sinus rhythm, and antiarrhythmic medication with Amiodarone was initiated. Coronary artery disease could be excluded by a coronary angiography, and subsequent left ventricle (LV) endomyocardial biopsies (EMBs) were performed. A guideline-directed heart failure therapy, including Dapagliflozin, Sacubitril/Valsartan, Eplerenone, and beta-blockade, was initiated. Further diagnostic work-up by cardiac magnetic resonance (CMR) imaging on day 8 after admission confirmed a severely reduced LV systolic function with an estimated ejection fraction of 18%, hypokinesia inferoseptal and mediobasal, and a mild LV hypertrophy with an intraventricular septal thickness of 14 mm. Stroke volume index was estimated with 25 ml/m^2^. CMR showed patchy subepicardial to transmural late gadolinium enhancement (LGE) anteroseptal, inferior, and inferolateral. Global native *T*1 and *T*2 were elevated with a *nT*1 of 1326 ms and a *T*2 of 60 ms (reference values: *nT*1 = 1256 (1240–1280) ms and *nT*2 = 45 (44–47) ms). LGE was evident also in the right ventricular wall. Additionally, enlarged mediastinal and hilar lymph nodes could be detected and a minimal left sided pericardial effusion (Figures 3(a), 3(b), 3(c), and 3(d)). Histology and immunohistology of the EMB revealed an acute lymphocytic myocarditis with focal myocyte necrosis and infiltration of CD3+ T cells and CD68+ macrophages on day 13 (Figures 4(a), 4(b), and 4(c)). Reverse transcription polymerase chain reaction (RT-PCR) for common cardiotropic viruses or bacteria was negative. Moreover, qRT-PCR analyses of the endomyocardial specimen for COVID-19 were negative. Thus, in absence of infectious agents in the myocardium, an immunosuppressive therapy was initiated on day 14 with prednisolone (1 mg/kg/day) and continued for 2 weeks, followed by a dose tapering regimen of 10 mg every 4 weeks in combination with azathioprine 300 mg/day. Due to a significant QTc interval prolongation, Amiodarone had to be terminated, and the patient was equipped with a LifeVest®. On day 17, a non-sustained ventricular tachycardia was documented (Figure 5). Heart failure therapy achieved remission of symptoms, and the patient could be discharged 18 days after admission. He was advised to refrain from high-intense physical activity. Follow-up CMR imaging, scheduled 3 months after hospital discharge, could demonstrate an improved LV systolic function with an estimated LV ejection fraction of 36% and a stroke volume index of 45 ml/m^2^. While native *T*1 and *T*2 normalized, strong LGE was still detectable. Combined immunosuppressive therapy with prednisolone in a dose tapering regimen in combination with azathioprine 300 mg/day was suggested to continue for 6 months.

## 3. Discussion and Conclusions

In this work, we present a unique case of acute lymphocytic myocarditis in a young man post-COVID-19. An association between a SARS-CoV-2 infection and myocardial injury has been suggested since the outbreak of COVID-19 [3]. The frequency and prognostic impact of COVID-19-mediated myocarditis are unknown [4]. In 2020, SARS-CoV-2 virions were detected in myocytes of infected patients revealing myocarditis [5]. Until 2021, a total of 38 cases (26 male, 24 aged <50 years) of myocarditis in confirmed COVID-19 patients were reviewed [4]. In this report, five patients died in hospital, and the first case was a virus-negative lymphocytic myocarditis, but myocarditis secondary to SARS-CoV-2 cardiotropism has also been demonstrated in EMB [4]. In an autopsy study of eight patients with severe COVID-19, active lymphocytic myocarditis was histologically found in all cases, although myocarditis was not clinically diagnosed [6]. In a clinical study from Wuhan by January 2020, myocardial damage was diagnosed in 12% among 41 admitted hospital patients (30 male) with confirmed COVID-19 [7]. Typical new onset of heart failure symptoms leading to admission in our patient in combination with elevated troponin level and severely reduced LV ejection fraction were suspicious for myocarditis in a young man [8, 9]. The definite diagnosis of lymphocytic myocarditis was established in EMB and underlines the recommended concept of EMB as gold standard in patients with clinically suspected myocarditis [10]. Lymphocytic myocarditis as presented here, however, could have a long list of potential causes, mainly virus-induced, by direct virus-mediated or indirect immune-mediated myocardial injury [11]. Persistence of the virus in EMB was associated with worse outcomes compared to viral clearance [12]. Of note, parvovirus B19 appears to cause both virus-mediated and virus-triggered myocarditis, whereas respiratory viruses, such as influenza and coronaviruses, can trigger an immune-mediated lymphocytic myocarditis in the absence of viral genome in the myocardium [13, 14]. However, cause–effect relationship between SARS-CoV-2 infection and myocarditis is difficult to demonstrate [4]. In our case, describing a young patient, recently going through COVID-19, we could not detect SARS-CoV-2 RNA by RT-PCR in EMB. This might, as discussed in a report of 18 patients with COVID-19 and myocardial injury, be explained by transient viral infection or due to immunologic effects causing myocardial inflammation without direct viral infection of cardiomyocytes [4, 15]. It is not uncommon that the underlying organism in lymphocytic myocarditis is not detected as it is often cleared by the immune system before significant inflammation occurs [2, 16]. In viral myocarditis, follow-up analysis of EMBs by PCR documented spontaneous clearance of viral genomes in 36.2% [12].

Acute myocarditis following mRNA COVID-19 vaccination is reported by the European Medicine Agency safety committee as a rare adverse event [17]. Also, according to the US Centers for Disease Control and Prevention, myocarditis/pericarditis rates are described with 13 cases per million doses of second-dose mRNA vaccine [18]. In multiple studies/case reports describing acute myocarditis following COVID-19 mRNA vaccines, however, this adverse event usually occurs shortly after the second dose [8, 19–21]. The patients presented with chest pain had elevated cardiac troponin levels, an abnormal ECG with ST elevations in most, and CMR imaging was suggestive of myocarditis in all and none of the patients had evidence of acute COVID-19 [21–23]. Typically, these patients are younger aged male and most required hospitalization up to 4 days but were considered mild [24]. Most cases recovered completely with or without heart failure treatment within 1 month [19, 21, 24–26]. Most recently it was shown that, especially in young men, IL-1RA antibodies are relevant in the pathogenesis of myocarditis after SARS-CoV-2 mRNA vaccination [27]. Despite rare cases of myocarditis, the benefit–risk assessment for COVID-19 vaccination showed a favorable balance for all age and sex groups [21].

As clinical condition was sufficiently stable in our patient, heart failure medication was initiated immediately [28]. The beneficial effect of immunosuppressive therapy in biopsy-proven virus-negative lymphocytic myocarditis could be demonstrated in the randomized, double-blind, placebo-controlled Tailored IMmunosuppression in virus-negative Inflammatory Cardiomyopathy (TIMIC) trial including 85 patients [29]. In this trial, the patients received prednisone and azathioprine (*n* = 43) vs. placebo (*n* = 42) for 6 months [29]. The benefit from immunosuppressive therapy could recently be demonstrated also after long-term follow-up (up to 20 years) [30]. The risk of cardiovascular death and heart transplantation was significantly lower in treated patients included in the TIMIC trial, and patients showed persistent improvement in the LV ejection fraction compared with the placebo group [30]. Long-term benefit of combined immunosuppressive therapy, including steroids and azathioprine, could also be shown in dilated cardiomyopathy [31]. Early and late favorable effects of immunosuppressive therapy could particularly been shown for improvement of LV function, although improvement was also shown in the placebo group, but less often [31]. In our patient, however, we thought improvement of LV function was most probably related to immunosuppressive therapy. Until now, limited data support the use of immunosuppressive agents in COVID-19-associated cardiac involvement [32]. In acute myocarditis, as in our case, polymorphic and irregular ventricular arrhythmias are particularly common and present a relevant risk [33]. As a consequence, a LifeVest®, as wearable cardioverter defibrillator for primary prevention of sudden cardiac, was implanted due to the high risk of sudden cardiac death in our patient. While the patient has improved LV ejection fraction >35%, was asymptomatic with remission of symptoms, NYHA I, and had no history of cardiac arrest or sustained ventricular tachycardia, no permanent cardioverter defibrillator was implanted. He had clearly a lowered risk due to heart failure therapy, and the European Society of Cardiology guidelines recommend an cardioverter defibrillator in either secondary prevention or symptomatic patients (NYHA II–III) with an LV ejection fraction ≤35% [33]. Physical activity should be restricted during the acute phase of myocarditis until the disease has completely resolved as recommended in our case [34].

## 4. Conclusion

The case presented highlights the importance of awareness for an extrapulmonary manifestation after COVID-19 involving the heart. There is need for evaluation of therapeutic approaches in acute lymphocytic myocarditis related to COVID-19 to facilitate the development of personalized treatment options.

## Figures and Tables

**Table 1:** 

Time	Events
Day 1	1-month history of increasing fatigue, palpitations, and shortness of breath
	12-lead electrocardiogram showed an atrial tachycardia with 127 bpm. Transthoracic echocardiogram showed a severely impaired biventricular systolic function with an estimated left ventricular ejection fraction of 18%. After excluding intraventricular thrombus formations, electric cardioversion could restore sinus rhythm.
Day 2	Coronary angiography showed normal coronary arteries. Left ventricular endomyocardial biopsy was performed.
Day 2	Transfer to intermediate care unit and optimization of medical heart failure therapy.
Day 8	Cardiac magnetic resonance imaging confirmed a severely reduced left ventricular function. Increased signaling of the interventricular myocardium and strong midmyocardial late gadolinium enhancement (LGE) was noticeable. LGE was evident also in the right ventricle.
Day 13	Histology and immunohistology of the endomyocardial biopsies revealed an acute lymphocytic myocarditis. RT-PCR revealed no infection with common cardiotropic viruses. Immunosuppressive therapy with prednisolone was initiated (1 mg/kg/day) in combination with azathioprine 300 mg/day.
Day 15	Patient was equipped with a LifeVest®.
Day 17	Documentation of a non-sustained ventricular tachycardia.
Day 20	Patient was discharged on a steroid taper in combination with azathioprine 300 mg/day.
Follow-up at 3 months	Follow-up cardiac magnetic resonance imaging was performed on an outpatient basis. An improved LV systolic function with an estimated LV ejection fraction of 36% was evident, and a strong LGE was still detectable.

## Data Availability

Data and material are presented within the manuscript.

## Abbreviations

EMB: Endomyocardial biopsy
LV: Left ventricle
COVID-19: Coronavirus disease 2019
CMR: Cardiac magnetic resonance
LGE: Late gadolinium enhancement.
